# Exploring lived experiences on the usage of removable complete dentures among edentulous patients attending Makerere University Dental Hospital, Kampala, Uganda

**DOI:** 10.1186/s12903-024-04484-3

**Published:** 2024-06-19

**Authors:** David Nono, Godfrey Bagenda, Isaac Okullo, Charles Mugisha Rwenyonyi

**Affiliations:** 1 School of Clinical Research, Central University of Nicaragua (UCN-Central Campus), Managua, Nicaragua; 2 School of Clinical Research, Texila American University, Georgetown, Guyana; 3 School of Dentistry, College of Health Sciences, Makerere University, Kampala, Uganda; 4 School of Biomedical Sciences, College of Health Sciences, Makerere University, Kampala, Uganda

**Keywords:** Edentulous patients, Informed consent, Oral function, Removable complete dentures

## Abstract

**Background:**

Edentulism remains a major disability worldwide, especially among the elderly population, although the prevalence of complete edentulism has declined over the last decades. In Uganda, the prevalence of edentulism in people aged 20 years and above is 1.8%. The therapy for edentulous patients can be realized through the use of conventional removable complete dentures, implant-supported prostheses, and computer-aided design and computer-aided manufacturing (CADCAM), however, the provision of removable complete dentures continues to be the predominant rehabilitation for edentulous patients. However, no published study has explored the lived experiences with removable complete dentures among the Ugandan population. The aim of the present study was to explore patients’ lived experiences on the usage of removable complete dentures among Ugandan edentulous patients attending Makerere University Dental Hospital.

**Methods:**

This was a qualitative study approach using purposive sampling. Fifteen (15) respondents were selected across social demographics. Interviews were recorded and transcribed and themes were generated to draw a deeper meaning to the usage of removable complete dentures. A qualitative statistical package, Atlas Ti software was used to generate themes from the interviews followed by an interpretation of the generated data and the results were presented as text and in a table.

**Results:**

The reported key positive experiences due to removable complete denture rehabilitation were the improvement in speech, eating ability, regaining good facial appearance, better oral hygiene management, self-esteem and confidence to smile in public, and a feeling of completeness. However, respondents complained of pain and discomfort due to the looseness of dentures, inability to eat certain foods, and regular cleaning of dentures. The respondents did not go through proper informed consent processes before getting removable complete dentures.

**Conclusion:**

The study found that patients were satisfied with their removable complete dentures rehabilitation due to the positive experiences registered, such as the ability to eat and talk well, and restoration of self-esteem, all of which improved their quality of life. However, they experience pain and discomfort due to the looseness of dentures.

**Supplementary Information:**

The online version contains supplementary material available at 10.1186/s12903-024-04484-3.

## Introduction

Edentulism (tooth loss) remains a major disability worldwide, especially among the elderly population, although the prevalence of complete edentulism has declined over the last decades [1–3]. In Uganda, the prevalence of edentulism among the population aged 20 years and above is 1.8% [4]. Total tooth loss contributes to disability, impairment, and handicap [5]. Replacement of the natural teeth and their associated parts by artificial substitutes can be achieved using a removable complete denture (RCD). At least in sub-Saharan Africa, oral health problems greatly contribute to morbidity and in the case of edentulous patients, it leads to the desire to obtain RCD [6–8]. RCD restores facial appearance and oral function, however, a good RCD depends on good interactions between the patient and dentist [9–12]. Apart from RCD, the therapy for edentulous patients can be realized through the use of implant-supported prostheses, computer-aided design, and computer-aided manufacturing (CADCAM), however, the provision of RCD continues to be the predominant rehabilitation for edentulous patients [13, 14].

The predominance of RCD is dictated by economic reasons, aesthetic acceptability, and ease of cleaning, and has been in use for a very long time with varying degrees of success [15, 16]. A complete denture is essential to rehabilitate the stomatognathic system by improving the masticatory efficiency, phonetics, social esteem, and aesthetic appearance of completely edentulous patients [17–19]. Restoring masticatory function is vital because it impacts food digestion and the patient’s quality of life []. However, it has been acknowledged that several people are experiencing some challenges with complete dentures [20].

The Uganda National Oral Health Policy [21] recommended the fabrication of conventional RCDs for the rehabilitation of edentulous patients using internationally accepted standards which include both clinical and laboratory procedures involving taking preliminary and final impressions, recording jaw relations using occlusal rim block (ORB), trial fitting of the wax denture, conversion of wax into the acrylic denture, and delivery or insertion of final RCD [22, 23]. However, the ORBs produced by this recommendation are not suitable for Ugandan patients [24], hence could be contributing to the post-insertion complaints associated with removable complete dentures [25]. These complaints include lack of retention and stability during function, pain or discomfort, accumulation of food under the denture, altered speech, unsatisfactory appearance, and gagging [26, 27].

It should be noted that wearing ill-fitting or uncomfortable complete dentures can have undesirable effects on the denture-supporting tissues, hence the oral health of the patient [28, 29]. Additionally, several studies have revealed different levels of experiences regarding the rehabilitation of edentulous patients which include the occurrence of blisters, difficulty in closing the mouth and loose dentures, painful gums, food lodgment, and unstable mandibular complete dentures as well as loss of retention and mucosal irritation [30–33]. However, no published study has explored the lived experiences with RCDs among the Ugandan population. Therefore, the aim of the present study was to explore patients’ lived experiences on the usage of RCD among Ugandan edentulous patients attending Makerere University Dental Hospital.

## Materials and methods

### Study design

The study employed a qualitative study design where data were collected through in-depth interviews.

### Study site

The study was conducted in Makerere University Dental Hospital, located in Kampala, the capital city of Uganda. The hospital is a teaching and health service delivery facility of Makerere University staff and students as well as the surrounding community. It is the largest and adequately equipped dental facility employing the highest number of oral health workers in Uganda. It has a well-established prosthetic dental laboratory that offers various services including rehabilitation of edentulous patients with RCD at a minimal fee. The hospital attends to approximately 660 outpatients per month of which about 20 are treated using RCD (Registry of Dental Records, 2022). It was chosen as a study site because of the large number of registered edentulous patients which could easily raise the required sample size.

### Selection of study participants

The number of respondents (*n* = 15) was determined based on data saturation. Data saturation occurred on the 15th participant because all the relevant concepts and information needed to draw the appropriate conclusions regarding lived experiences with complete dentures had been obtained. Therefore, gathering additional data would not produce any new codes or insights. The respondents who had worn RCD for at least one month were assumed to have adequate experience and were purposively selected for the study. Both male and female patients of different age groups were included to ensure gender balance in respondent selection.

### Inclusion criteria

The study included complete denture wearers who were aged 18 years and above, had worn complete denture(s) for at least 1 month, and were willing to give written informed consent to participate in the study.

### Exclusion criteria

Those who were unavailable during the period of data collection.

### Data collection procedure

Before participating in the study, written informed consent was requested from the respondents. Patients who came for dental treatment and have worn removable complete dentures (RCDs) for more than one (1) month were asked to take part in the in-depth interview (IDI) using an interview guide (Appendix 1) to explore their lived experiences on the usage of RCD. The investigator explored patients’ views regarding causes of tooth loss, experience of being edentulous, oral functions (like eating, speaking, smiling, laughing, and talking), maintaining oral hygiene, benefits of wearing RCD, informed consent process, coping ability with RCD, decision to acquire RCD, maintenance and cleaning RCD. The in-depth interview of each respondent involved taking notes and audio recordings for 30 to 45 min by a trained research assistant who is a social scientist with experience in qualitative research methods. Additional notes on body language and gestures were also taken. The principal investigator counter-checked the notes and audio recordings for errors and completeness.

### Quality control

To ensure the collection of good quality data: (1) data collection tools were pretested by the principal investigator and amendments were made to improve their validity and reliability (2) two research assistants were trained in data collection techniques and (3) all IDIs were audio-recorded using the functional device that was verified before the start of the interview.

Credibility, Confirmability, Dependability, and Transferability were used to guarantee trustworthiness.

Peer debriefing and enlisting the assistance of more experienced qualitative researchers to review and provide feedback on the study procedure and findings to guarantee that the data is correct and pertinent helped to establish credibility.

#### Transferability

A thorough explanation of the research context, including the features of the chosen participants and setting, is given in the methods section. Readers are advised to utilize this description to determine whether or not they can apply the findings to their contexts or settings.

#### Dependability

A detailed description of the study procedures and analysis was provided, enabling its replication.

#### Confirmability

To ensure that the research study’s conclusions are based on the participants’ narratives and words and free from bias, a clear coding scheme was utilized to generate codes and uncover trends in the analysis.

### Data management and analysis

Transcription of the audio recordings was handled by a person experienced in qualitative research methods and grammar mistakes were also corrected. This was to ensure that good quality information is collected and is also kept in context. All soft copies of the transcripts were backed up on an external device and in space (Google Drive) with password protection, while a hard copy was kept under lock and key. A thematic-data analysis approach was used. A qualitative software package, Atlas Ti was used to generate themes from the interviews followed by an interpretation of the data, and the results were presented as text and in a table.

### Ethical consideration

Ethical approval of the protocol was obtained from the Makerere University School of Health Sciences Research Ethics Committee (Reference Number: MAKSHSREC-2023-486) as well as the Uganda National Council for Science and Technology (Reference Number: HS3092ES). Permission to carry out the study was obtained from the administration of Makerere University Dental Hospital. Written informed consent was obtained from all the respondents who took part in the study. The purpose of the study was explained to the respondents and their participation was voluntary following the Helsinki Declaration [34]. All the data collected were securely kept in a cabinet under lock and key and only accessible to the investigators.

## Results

Of the 15 respondents interviewed, the majority were females (*n* = 8, 53%). Most respondents (86.7%) were aged 41 years and above. A third (33.3%) of the respondents had attained secondary school and tertiary education. More than half (53.3%) of the respondents were married. Nearly half (46.7%) of the respondents were involved in farming activities. More than half (53.3%) of the respondents had been wearing RCDs for less than one year (Table 1).

**Table 1:** Demographic characteristics of the respondents

Variable	Frequency (*N* = 100)	Percentage (%)
**Gender**		
Male	7	46.7
Female	8	53.3
**Age**		
Less than 30 years	1	6.7
31-40yrs	1	6.7
41-50yrs	7	46.7
51 and above	6	40.0
**Education**		
No formal education	1	6.7
Primary	4	26.7
Secondary	5	33.3
Tertiary	5	33.3
**Marital status**		
Single	7	46.7
Married	8	53.3
**Occupation**		
Self-employed	10	66.7
Formally employment	3	20.0
Other	2	13.3
**Duration of wearing dentures**		
1 month-< 1 year	8	53.3
1–5 years	1	6.7
More than 5 years	6	40.0

### Themes from the study

This study produced four themes, which are as follows: physical experiences with wearing RCDs, emotional experiences with wearing complete dentures, experience with oral health while wearing RCD, and experiences with RCD care and maintenance.

#### Physical experiences with wearing RCDs

In understanding the respondents’ physical experiences with wearing RCDs, responses were captured regarding; the speaking and eating experiences after receiving dentures, the oral health effects of dentures, the physical adaptation to changes brought by wearing dentures, and experiences with denture care and maintenance.

##### The eating experiences

When the respondents were interviewed about their eating experiences when they started wearing dentures, it was found that most of them were having trouble with the choice of food to eat while wearing dentures. They had to select soft foods like rice and bread. Sometimes they felt discomfort as if dentures were coming out while eating. There are certain foods they had given up eating completely, such as popcorn, cassava, sugar cane, grasshoppers, and roasted ground nuts, among others because these are difficult to eat with the dentures, as they could experience gum pain while eating. Therefore, respondents had to be cautious with their dentures and avoid very hard foods. However, respondents could eat soft foods like posho, rice, sweet potatoes, beans, and meat if well-cooked. However, sometimes even while chewing soft food, it would get stuck in the denture teeth. Some of the respondents mentioned that they never used dentures to eat any food; rather they used the few remaining natural teeth in their gum for eating and only put on the dentures to move around. The details are given below:

*“There are some things I just gave up on. Like popcorn, cassava, or food that is so hard that requires me to strain a lot, I don’t eat it. But I eat soft food like posho, rice, sweet potatoes, beans and bananas. Bread is a bit of a challenge when you’re having tea”* (*P006_Male*).

*“I eat, but not hard stuff, I don’t eat hard cassava yet I love it. I have to eat foods like matoke, which is soft, I don’t eat meat and can no longer taste the roasted ground nuts”* (*P010_Female*).

##### The speaking experiences

In understanding the speaking experiences resulting from wearing dentures, it was found that some people were unable to speak clearly. Some mentioned that they usually speak well with dentures, but there were instances where they struggled if dentures were loosely in contact with the gum or some other changes in their mouth. Others highlighted that when the dentures are inserted properly into the gum, speaking was always okay and seems normal. However, some respondents explained that even when dentures were worn properly, speaking was a challenge, and their audience would always say that they could not hear them properly whenever they spoke. Some of the respondents’ accounts are given below:

*“When it is inserted firmly, oh you talk properly. But when it plays around, you lose fluent speech”* (*P001_Male*).

*“When I am putting on dentures, I have a problem with speech actually. People say they can’t hear me properly”* (*P009_Male*).

Whereas respondents reiterated several speaking challenges encountered with wearing dentures, some of them expressed that wearing dentures did not affect their speaking in any way. Some of the respondents’ statements were captured:

*“I have no challenge in speaking. I can speak and you can hear me properly”* (*P002_Male*).

#### Emotional experiences with wearing complete dentures

##### Emotional changes experienced with wearing RCDs

The study found that respondents were happy with their new teeth because they felt comfortable and that dentures had positively changed their appearance beyond their imaginations. The respondents mentioned that, despite the embarrassing moment of having to remove dentures when it’s time to dine with other people, dentures restored their self-esteem because their hitherto sunken jaws were now restored and they could speak well in public and smile without fear of being despised. They also mentioned the positive impact it had on their communication with others, as people no longer despised them whenever they spoke. Some respondents said:

*“The change is positive. The dentures changed my appearance because the jaws had sunk in, I was looking older than my age and I was not feeling comfortable. But now, as you can see, I look normal and my appearance is beautiful”* (*P004_Male*).

*“It makes me look comfortable and I can smile. I am just happy and I thank God that I was able to get this because I didn’t expect it”* (*P001_Male*).

##### Social interactions while wearing RCDs

Respondents were interviewed about how wearing RCDs affected their interactions with other people. Results showed that the people with dentures were freely interacting with other people without any form of discrimination. They always attended functions, but nobody would notice that they wore dentures. They explained that denture-wearing has not affected their relationships, and a few people who knew about their situation even congratulated them. In most cases, only a few close family members know about it, and the general public would not notice that they wore dentures unless they revealed it themselves. Friends were okay with it and at some point, they had also expressed interest in dentures. Having dentures has allowed them to laugh freely and they are no longer scared or self-conscious. Some of the respondents intimated:

*“I don’t have any problem, as you can see, I have my business that sells drinks, but I don’t have any problem. Like I told you, no one can know apart from those that I told I have a denture. Some even congratulate me”* (*P008_Female*).

*“My social life changed a lot because now I can laugh at funny stories and jokes. But by then, I would hold a cloth and cover my mouth. I was scared, but now that I have them I laugh well with my friends”* (*P015_Female*).

#### Experience with oral health while wearing RCD

The study found that the respondents who wear dentures face challenges with eating and cleaning them because they frequently remove and clean the dentures to prevent food from getting stuck. But overall, the dentures had improved their oral hygiene because they would clean their mouth and dentures at least twice a day, compared to when they would clean their mouth once or even not at all for a whole day. This was after denture treatment where the doctors advised them to take good care of their oral hygiene as much as they take care of the dentures. The respondents further explained their experiences as shown below:

*“At times, I eat, but as a person with a denture I feel that the food gets clogged, so I have to remove the denture, clean it put it back in the mouth”* (*P008_Female*).

Whereas the respondents’ regular cleaning had resolved issues with bad odor, they experienced some oral health challenges with dentures. Respondents explained that they experienced discomfort, wounds, and pain while chewing. Some of them had their gum changed slightly due to prolonged use of the dentures. Some of the respondents’ narratives were recorded:

*“As you know I have overused them in the gum, the gum has changed a bit from the way it was. You can see that where their denture sits changed a little”* (*P012_Female*).

*“After getting dentures, I had some small swellings on the gum, so I feel pain while chewing”* (*P004_Male*).

#### Experiences with RCD care and maintenance

##### Cleaning and maintenance of RCDs

The study found that respondents cleaned their dentures at least twice a day using lukewarm water with soap, while others used just a piece of cloth without toothpaste. They removed the dentures at night when they were going to sleep, cleaned and stored them in a cup with cold water, while others kept them in warm water. They also cleaned dentures in the morning before wearing them back. Respondents explained that they were advised by the doctors to clean the dentures with water and soap, but not toothpaste. Some of the respondents reported:

*“I clean the dentures using a piece of cloth by rubbing them. I don’t brush them using a toothbrush. I wake up every morning and just rub them with a piece of cloth. Then I rinse the mouth and wear the dentures”* (*P009_Male*).

*“I clean it [denture] in the morning and in the evening. I use hot water to clean them and then I use toothpaste to brush. I remove them at night when I am going to sleep and place them in a cup full of cold water”* (*P001_Male*).

*“I was told that after removing it from the mouth, I have to keep it completely immersed in water. So, that is what I do. I keep it in cold water and then brush it with cold water before wearing them every day”* (*P015_Female*).

The respondents generally reported that they followed the dentists’ instructions regarding their dentures. The majority of the respondents believed that they followed the dentists’ instructions at least around 80% of the time.

*“I follow the doctor’s instructions. I can give myself 80% of compliance”* (*P001_Male*).

*“I follow the doctors’ instructions to remove them [dentures] from the mouth before sleeping.”* (*P003_Female*).

##### Challenges with RCDs cleaning and maintenance

The study respondents acknowledged the importance of taking care of dentures by cleaning them regularly, but they expressed the latter as tedious and difficult to comply with. Moreover, the dentures had to be cleaned before putting them back in the mouth, which they expressed as creating an extra task that they were not accustomed to. One respondent explained:

*“I have to take care of it [denture] as I have to clean it now and then, which is tedious”* (*P001_Male*).

Respondents also explained that they sometimes have trouble getting their dentures properly cleaned no matter how much they try to clean them and suspect it could be due to not brushing them properly. It is challenging for them to maintain the necessary extra care of cleaning the teeth that are detached from the mouth. This was shown in some of the respondents’ narratives:

*“They require when you are extra clean. If I eat then I have to brush them too much. If I spend like three days or a day without brushing, they fade and cannot be as white as natural teeth. Now, I brush a little more every after eating” (P011_Male).*

Other respondents were unsure about the safety of the water they use to clean their dentures. They suggested using boiled water, although not all of them had access to clean and safe water at all times especially when they are at the workplace or generally away from home. Therefore, respondents expressed concern about the potential risks of using un-boiled water on dentures, especially when at the place of work, which may cause water-related infections. One respondent articulated:

*“Tthere are times when I am at the place of work and when it is time for meals, I remove and I put it in tap water or water from the jerry cans that I am not sure whether they are clean. Removing and fixing it in my mouth, I fear that at one point I may be affected with that water because it is not boiled” (P004_Male).*

Some of the respondents, however, were finding it convenient to clean their dentures after every meal and it did not bother them, because they were aware that it was for their health benefits.

*“I find no problem with following the doctor’s instructions to clean my dentures because it is to help me” (P007_Female).*

##### Dealing with denture cleaning challenges

Respondents mentioned that they had to get used to the dentists’ recommendations of cleaning their dentures and the mouth several times than they used to do, and to always remove and keep their dentures in containers with clean water. To deal with the risk of cleaning dentures with unclean water, some of the respondents highlighted that they often pack clean boiled water from home which they use to clean their dentures and the mouth while at workplaces. Some respondents mentioned that they have multiple dentures that they pack in their bags upon leaving their homes such that they had options to change the dentures whenever they needed to:

*“Whenever I am leaving home for work or going anywhere, I pack my boiled water. Sometimes I buy mineral water and use it to clean my dentures when it is necessary and if I am not at home”* (*P003_Female*).

*“I usually change, different dentures. I have many, so I keep some in my bag and I keep changing on different occasions”* (*P013_Female*).

## Discussion

This study explored the patients’ lived experiences on usage of RCD among patients. Despite the limitation of drawing purposively selected respondents from a single health facility, the present study established the baseline data of the lived experiences in the usage of RCD among edentulous patients in Uganda.

Despite a few respondents who experienced initial challenges in eating with RCDs, most of them (who happened to be 41 years above) were emotionally appreciative of the RCD treatment. This finding corroborates another study [35], which reported that the need to replace teeth becomes nearly universal as people become older. A study [36] done in the United States also found that many people get their first set of RCD between 40 and 49 years old.

The analysis showed that the course from being toothless to wearing complete dentures involved a mixture of positive and negative experiences. From the positive perspective, dentures improved the wearers’ Eating Related Quality of Life whereby they resumed eating some of the foods that they had given up when they were toothless such as peanuts. This is in accordance with the results of previous studies [37], which found that wearing dentures improves people’s dietary intake due to their regained ability to eat certain food items that they could not easily consume when they were toothless. Moreover, the present study found that wearing dentures restored the respondents’ confidence to eat in public without any worries of possible mockery, which is in accordance with the results from earlier studies [32]. These findings postulate that wearing dentures not only improved the patient’s self-esteem and ability to socialize but also their dietary intake and possibly improved their nutritional status. The result implies that edentulous people ought to improvise with dentures or any other credible form of prosthetic rehabilitation to improve their self-consciousness, diet, and subsequent healthy lifestyle.

The results further indicated that wearing dentures also improved the patients’ Oral Health because the denture wearers who had become reluctant to brush toothless gum were now encouraged to clean dentures and the remaining natural teeth on a regular basis at least every after a meal and before going to sleep. Related studies also reported a positive impact of wearing dentures on the Oral Health-Related Quality of Life (OHRQoL) [38, 39]. It is apparent that cleaning a mere gum even when it is only missing the anterior teeth can be challenging because one needs to use a cloth to clean a bare gum but also use a toothbrush and toothpaste to blush the few surviving teeth.

The present study found many other positive experiences that relay the respondents’ satisfaction with denture wearing and these concur with the earlier studies [40], which reported improvement in the patient’s speech and public interactions. Rodrigues, similarly, to other workers [32, 41] reported that dentures helped to restore the patient’s smile, self-esteem, and a feeling of completeness. Based on these results, it is clear that the decision to wear complete dentures provided an alternative for the edentulous people especially, to regain full confidence in their lifestyles. Moreover, the respondents in the present study recommended that people who have a similar problem of toothlessness should consider going for denture rehabilitation due to the potential benefits it holds.

From the perspective of the negative experiences, the present study identified some complaints, especially with the post-denture insertion experiences, which were quite problematic to the patients. Pain and discomfort were reported as the common complaints of the complete denture wearers. A similar result was reported by other scholars [25, 42, 43]. In addition, Ubaid Iqbal and Lone [25] warn that having poor ridge contour and technical errors during RCD fabrication could result in loss of retention of RCDs. Furthermore, other complaints that expressed discomfort were; difficulty in eating and the limitation in the type of food the patients have to eat with RCDs, being unable to get the expected taste of food whenever they eat with dentures, clogging of food under and around the dentures which calls for regular denture and mouth cleaning every after a meal, and difficulty in speech worsened by the looseness of the dentures which creates a constant worry that dentures could fall-off from the mouth during any oral action. Similar studies also found that complete dentures compromised the wearers’ quality of life in terms of eating speaking, and oral hygiene maintenance [35, 42, 43].

Furthermore, several studies that are in line with the present study have revealed different levels of experiences regarding the rehabilitation of edentulous patients which include the occurrence of blisters, difficulty in closing the mouth and loose dentures, painful gums, food lodgment, unstable mandibular complete dentures as well as loss of retention and mucosal irritation [30–33]. This could explain the reason why most of the respondents tend to remove dentures and eat with their few surviving natural teeth or bare gum. Some of the respondents consider dentures as only a decoration of the mouth, which only serves to restore one’s facial beauty and self-esteem but did not help to improve quality of life in other ways.

In addition, among respondents’ complaints, physical pain peaked at the highest level which involves others like aching, soreness, and uncomfortable to eat. This finding is in line with a study done by Adam [44]. This is followed by another criticism which is the functional difficulties (87%), mastication, and catching food capacity. Some respondents felt unable to function. This finding agrees with other studies that correlate the functional limitation to the discomfort experienced by edentulous patients, the feeling of an unfitted denture, and food accumulation under dentures [2, 5, 45–47]. Many authors have reported that the design faults and the structural defects of the dentures are the prime cause of the complaints [25, 43, 48].

Therefore, addressing such complaints is a question of ensuring that the denture fabricators are well trained to take proper alignment of the complete dentures in a manner that they fit well onto the gum. Also, one scholar advises that; for proper basic considerations when establishing correct occlusion for complete dentures, professionals should ensure that the occluding rows of artificial teeth will provide optimum chewing efficiency, functional stability of the prosthesis, and comfort during chewing without exerting injurious forces on the denture-bearing tissues or adversely affecting aesthetics or phonetics [48]. It is also important that denture wearers endeavor to go for denture replacement, to avoid overuse which may damage the gum.

## Conclusion

After receiving dentures, patients expressed satisfaction with their decision to undergo denture rehabilitation due to the positive experiences registered, such as the ability to eat and talk well, and restoration of self-esteem, all of which improved their quality of life. However, they experience pain and discomfort due to the looseness of dentures and worry about the embarrassment when they fall off in public, the inability to eat all food as desired, failure to obtain food taste, and the burden of cleaning dentures so often.

### Implications for clinical practice

Findings from this study could help to inform dental technologists and dentists about the challenges faced by RCD wearers, and it could act as a point of reference for future denture fabrication and rehabilitation procedures. Furthermore, the findings can be used to review the clinical training curriculum for dental surgery and dental technology students.

### Implications for future research

Taking into account the results of the present study, the common negative experiences impinge around pain and discomfort due to the looseness of the dentures. Therefore, future studies could focus the attention on understanding the issues resulting in looseness of the dentures, and any other causes of oral discomfort among denture wearers. This could provide information for planning better technologies for RCD fabrication and extensive denture rehabilitation for effective clinical practice and to further enhance OHRQoL.

### Limitations of the study

Considering that edentulism is a systemic chronic disease common in the population, the selection of one study site (Makerere University Dental Hospital) and only 15 respondents may not give a better representation of the edentulous population. This study relied on the self-reporting of respondents and could be liable to response bias.

## Recommendations

There is a need for designing continuous professional development courses for dental practitioners and encouraging them to often undertake these courses to refresh their dental care and/or denture fabrication skills, improving the technology of RCD fabrication, training the denture wearers on denture maintenance and oral hygiene. These recommendations are crucial for the improved and excellent management of edentulous patients using RCDs.

### Electronic supplementary material

Below is the link to the electronic supplementary material.

Supplementary Material 1

## Data Availability

Data sources are available on request. The request can be sent to the corresponding author at nndvd45@gmail.com.

## Abbreviations

RCDs: Removable complete dentures
ORBs: Occlusal rim blocks
