# Supplementary material for: Exploring lived experiences on the usage of removable complete dentures among edentulous patients attending Makerere University Dental Hospital, Kampala, Uganda
Source: BMC Oral Health. 2024 Jun 19;24:709. doi: 10.1186/s12903-024-04484-3 (PMC11186153; doi:10.1186/s12903-024-04484-3)
Supplement: Supplementary file 1 — Supplementary Material 1 [file 12903_2024_4484_MOESM1_ESM.docx]

## Appendix 6: Interview Guide for Patients

**Study Title:** Estimation of standard height of occlusal rim block for complete denture fabrication among Ugandan Population in Makerere University Dental Hospital in Kampala, Uganda: Knowledge, Experiences, and Perceptions of Patients and dental practitioners

**Socio-demographics**

**Age (in years)** ………………………………………………………..…………………

**What is your sex?**

| Male |  |
| --- | --- |
| Female |  |

**Marital status:**

| Single |  |
| --- | --- |
| Married |  |
| Divorced/separated |  |
| Widow/Widower |  |

**Highest level of education**

| Informal education |  |
| --- | --- |
| Primary |  |
| O-level |  |
| A-level |  |
| Tertiary |  |

**Occupation**

| Unemployed |  |
| --- | --- |
| Self-employed |  |
| Formal employment |  |

**Experience during teeth loss**

1. Tell me when you started experiencing tooth loss? (What was the cause of tooth loss?)
2. How did you feel when you were completely edentulous? How were you faring without teeth or how did it affect you?
3. Tell me about your experience of being edentulous. Probe: How about your quality of life since you lost your teeth? Did you feel complete or not? How did being edentulous affect your self-esteem, your social life, and your being comfortable in social gatherings? Did you feel shy while in social gatherings? How did you cope with tooth loss?
4. How has living with this edentulous status affected your oral functions like eating, speaking, smiling, laughing, and talking to people?

**Knowledge of complete dentures**

1. How did you come to learn about complete denture treatment? (Who informed you, where did you read about it)
2. What do you know about complete dentures?
3. What are the benefits of wearing complete dentures?
4. What are the challenges associated with complete dentures

**Perceptions of complete dentures**

1. What does having complete denture(s) mean to you?
2. How do you feel to wear denture(s) in comparison to natural teeth?
3. When wearing complete dentures, do you think people you interact with are likely to know you have artificial teeth? What do they think when they get to know or what do they say?
4. When they get to know that you are wearing denture(s), what effect does it have on your self-esteem? How do you feel when you are in a gathering? Do you feel shy, do you feel small?

**Experience with complete dentures**

1. Please tell me about the tooth replacement with complete dentures. Probe: When did it start and what was the process you went through?
2. What is your experience in wearing dentures (Probe: Positive and negative experience, the benefits and challenges)
3. What challenges did you face during denture rehabilitation?
4. How did you cope with the complete denture rehabilitation process?

Probe:

1. What strategies are you adopting in order to ensure you wear your dentures? What helped you cope with the dentures?
2. Are these strategies helping you in overcoming the challenges you face while wearing your complete dentures?
3. Generally, how would you describe your experience throughout the complete denture treatment?
4. What foods do you usually like to eat? Probe: Are you able to eat food you used to eat or food that you like?
5. Please describe the challenges faced regarding wearing complete denture prostheses.
6. How are these challenges interfering with the daily wearing of complete denture prostheses?
7. What was the effect of complete denture treatment on your oral health?
8. How has living with the complete denture prostheses influenced your interaction with friends, interaction with other people, and being in public gatherings like parties, or other social events?
9. Has wearing complete dentures affected other aspects of your life?
10. How did you cope with the treatment procedure or other challenges you experienced?
11. Did you receive any form of counseling or support from your relatives, your family, or friends for complete denture rehabilitation?
12. Do you feel like what you went through denture treatment has changed you as a person?
13. What was your experience with the quality of services that were provided to you? (What didn’t you like about the treatment or the health care providers? What was not done well? What do they have to improve?)

**Experience with care and maintenance of complete dentures**

1. Please describe how you clean and maintain the dentures to ensure that they are hygienic.
2. Describe your level of compliance with the dentists’ instructions on cleaning and maintaining dentures (Rate your level of compliance).
3. What challenges are you facing regarding compliance with those instructions? (Probe for financial challenges, challenges in cleaning dentures

**Complete denture Information and communication with dental professionals**

1. What was your experience with the consent process during complete denture rehabilitation (Probe: what did they explain to you? Were you given a consent form to read? Were you given a verbal explanation or did someone read it for you?

What were you told or informed about complete dentures? Benefits or risks after receiving the dentures)

1. In your view, were you given adequate information about complete dentures? Is there anything they did not explain to you? Were you able to understand the information they gave you? Is there anything you did not understand?)
2. After the therapy did the dentist provide you with additional information on how to maintain the dentures that you were given?
3. In your view, what can be done to improve the patients’ experience during complete denture rehabilitation so that they don’t find any hardships?
4. Is there anything else you would like to add to what we have discussed?

**Thank you**
